# Galvanic Deposition of Pt Nanoparticles on Black TiO_2_ Nanotubes for Hydrogen Evolving Cathodes

**DOI:** 10.1002/cssc.202101559

**Published:** 2021-10-06

**Authors:** Aikaterini Touni, Xin Liu, Xiaolan Kang, Patricia A. Carvalho, Spyros Diplas, Kevin G. Both, Sotirios Sotiropoulos, Athanasios Chatzitakis

**Affiliations:** ^1^ Department of Chemistry Aristotle University of Thessaloniki 54124 Thessaloniki Greece; ^2^ Centre for Materials Science and Nanotechnology Department of Chemistry University of Oslo Gaustadalléen 21 0349 Oslo Norway; ^3^ SINTEF Industry POB 124 Blindern 0314 Oslo Norway

**Keywords:** electrocatalysis, electrode materials, hydrogen evolution, Pt electrocatalyst, TiO_2_ nanotubes

## Abstract

A galvanic deposition method for the in‐situ formation of Pt nanoparticles (NPs) on top and inner surfaces of high‐aspect‐ratio black TiO_2_‐nanotube electrodes (bTNTs) for true utilization of their total surface area has been developed. Density functional theory calculations indicated that the deposition of Pt NPs was favored on bTNTs with a preferred [004] orientation and a deposition mechanism occurring via oxygen vacancies, where electrons were localized. High‐resolution transmission electron microscopy images revealed a graded deposition of Pt NPs with an average diameter of around 2.5 nm along the complete nanotube axis (length/pore diameter of 130 : 1). Hydrogen evolution reaction (HER) studies in acidic electrolytes showed comparable results to bulk Pt (per geometric area) and Pt/C commercial catalysts (per mg of Pt). The presented novel HER cathodes of minimal engineering and low noble metal loadings (μg cm^−2^ range) achieved low Tafel slopes (30–34 mV dec^−1^) and high stability in acidic conditions. This study provides important insights for the in‐situ formation and deposition of NPs in high‐aspect‐ratio structures for energy applications.

## Graphical abstract

**Be spontaneous**: Pt nanoparticles of a diameter of around 2 nm are deposited on black TiO_2_ nanotubes via the spontaneous galvanic deposition process, across the whole nanotube length truly utilizing the structure's high‐aspect‐ratio surface area. These electrodes of minimal engineering and low noble metal loadings show high activity and stability towards the hydrogen evolution reaction in acid.

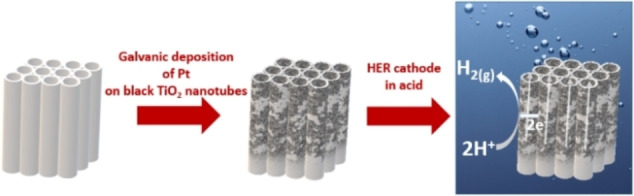

## Introduction

Recent studies, mainly carried out during the last ten years, reflect the unique properties of black titania. Titania is a semi‐conducting material with a large bandgap (3.2 eV for anatase), and its properties include earth abundance, non‐toxicity, and chemical stability in different media.[^1^, ^2^, ^3^] In contrast to the pristine TiO_2_, black titania shows metallic‐like electronic conductivity,[^2^, ^4^, ^5^] which makes it an attractive oxide material for electrochemical investigations. Its dark color assists the adsorption of sunlight^[6]^ in a wide wavelength range, from ultraviolet up to the near‐infrared region,[^7^, ^8^] due to the narrow bandgap and the mid‐gap electronic states.^[9]^ This material has gained great attention in the field of photocatalysis for the production of H_2_ gas during water splitting[^3^, ^4^, ^10^, ^11^, ^12^, ^13^] and decomposition of organic pollutants such as phenol,^[14]^ toluene,^[15]^ ethyl acetate,^[15]^ and methyl orange^[16]^ in wastewater treatment. Except for its importance in the field of photocatalysis, its use expands to a wide range of applications in the field of electrochemistry, such as lithium‐ and sodium‐ion batteries,[^3^, ^4^, ^17^, ^18^] supercapacitors,^[19]^ and fuel cells.^[20]^

Various synthetic routes have been reported to synthesize nanostructured black titania,[^2^, ^4^, ^21^] such as hydrogenation (H_2_, H_2_/Ar, H_2_/N_2_ at high/ambient pressure, H_2_ plasma),[^18^, ^22^, ^23^, ^24^, ^25^, ^26^, ^27^] chemical reduction (by Mg, Zn, Li, Al, NaBH_4_, CaH_2_),[^5^, ^11^, ^13^, ^15^, ^28^, ^29^] laser ablation/pulsed laser ablation, and electrochemical reduction.^[22]^ Also, microwave irradiation, ultrasonication,^[30]^ and one‐pot gel combustion have been reported in the literature[^2^, ^3^, ^21^] for the production of black titania.

In this work we utilized CaH_2_ to chemically reduce TiO_2_ nanotubes (TNT) in order to synthesize electronically conducting oxides of TNT (bTNT), which can then act as a support for Pt nanoparticles (Pt NPs) and the resulting electrode as an efficient cathode for the hydrogen evolution reaction (HER). Despite porous electrodes suffering from pore clogging when used in gas‐evolving electrochemical reactions, they are often employed for such reactions since their large electroactive area offsets gas blanketing effects (see for example the use of porous/nanoporous Ni electrodes as hydrogen evolution cathodes in alkaline water electrolysis^[31]^ or in electrochemical hydrogenation reactions^[32]^).

Although Pt/C powder catalysts adhered on the polymer electrolyte membrane is the common HER cathode choice in polymer electrolyte membrane (PEM) electrolyzers,^[33]^ there are a number of other industrial applications whereby platinized Ti electrodes are used as HER cathodes; these include, for example, electrooxidation of organics^[34]^ and photoelectrochemical water splitting.^[35]^

Our previous studies showed that highly oriented anatase bTNTs towards the [001] direction can be obtained when the CaH_2_ is in contact with the material.^[5]^ This is particularly important as the electronic conductivity of the material is significantly enhanced, a property that is highly desirable if it is to be used as an electrode support. Pt is the state‐of‐the‐art electrocatalyst for the HER in acidic media based on the relevant volcano plot.[^36^, ^37^, ^38^] In industrial electrolysis/photoelectrolysis and electrodialysis applications (where stable cathodes and anodes are needed, able to perform at high current densities), Pt particles are usually dispersed on a durable Ti‐based support. The 1D orientation of bTNT exhibits orthogonality to light absorption^[26]^ and provides high surface area,^[11]^ which is desirable for the dispersion of electroactive nanoparticles, likely to lead to improved performances for the HER at low mass loadings. On the other hand, the high aspect ratio of these nanostructures confines the majority of the surface area and the deposition of NPs along the entire length of the nanotubes is not straightforward.

Pt deposition on various black titania nanomaterials have been reported in the literature. Zhang et al.^[20]^ electrodeposited Pt on hydrogenated TNTs (H‐TNTs) and used both as anode and cathode at polymer exchange membrane fuel cells (PEMFC). Wang et al.^[39]^ prepared monodisperse Pt nanoparticles on N_doped_‐black TiO_2‐*x*_ by a borohydride reduction method and tested them for the HER and oxygen reduction reaction (ORR). Li et al.^[40]^ synthesized Pt/TiO_2‐*x*_ microspheres and used them as photocatalysts. Recently, Wang et al^[41]^ photo‐deposited Pt on black TiO_2_ powder for H_2_ evolution, and Wei et al.^[42]^ studied the HER on oxygen vacancy‐rich ($v_{O}$ ‐rich) and oxygen vacancy‐deficient ($v_{O}$ ‐deficient) TiO_2_ support deposited with Pt nanoclusters. Last but not least, other precious metals that have been deposited on black titania include Rh for HER (by a sputtering method proposed by Szenti et al.^[43]^) and Pd for ORR (on Al reduced‐TiO_2‐*x*_ nanobelts via the borohydride method proposed by Yuan et al.^[44]^).

Herein and for the first time we take advantage of the metallic conductivity of oriented bTNTs and we propose the galvanic deposition/replacement method to deposit Pt particles on its surface. The galvanic deposition process takes place spontaneously by mere immersion of the bTNT in a Pt(IV) complex ion solution, which makes it an easy and simple way to prepare platinized bTNT. It should also be stressed that the galvanic deposition is expected to lead to low loading deposition of the precious metal on the surface of the substrate and may also allow the formation of Pt NPs in the interior of the nanotubes. This is because Pt ions are in the liquid solution, which can flood the pores of the nanotubes and form Pt deposits that are otherwise impossible for such high aspect ratio structures. Electrocatalysts prepared by the galvanic deposition method are reported by Papaderakis et al.^[45]^ Lately, the galvanic deposition method has also been used by Schmuki and co‐workers^[46]^ to deposit metallic Ir on black titania for the HER, exhibiting high electrocatalytic performance.

The aim of this work has been the establishment of a simple galvanic deposition method to prepare metalized bTNT to be used as electrocatalytic materials. Its objectives have been: (i) preparation of Pt/bTNT by immersion of freshly CaH_2_‐reduced bTNT in a platinum chloro‐complex solution; (ii) microscopic and spectroscopic characterization of the material with emphasis on Pt insertion into the pores and potential metal‐substrate interactions; (iii) electrochemical characterization of the material via typical H adsorption/desorption and hydrogen evolution reactions; and (iv) elucidation of the deposition mechanism by density functional theory (DFT) calculations.

## Results and Discussion

### Structural characterization

**Figure 1:**
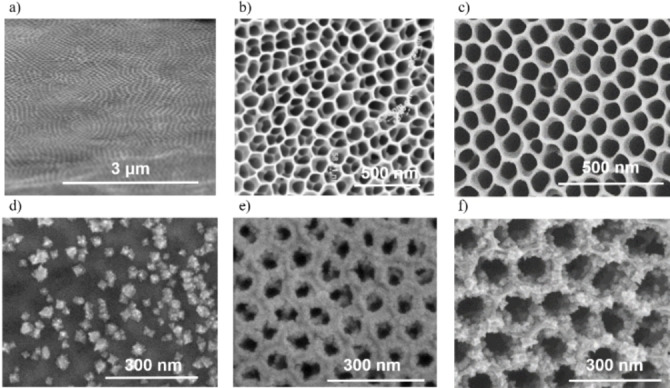
Top surface SEM images of (a) bTNT0, (b) bTNT5, and (c) bTNT30, before and after galvanic deposition of Pt in a solution containing 0.5 mm K_2_PtCl_6_ and 0.1 m HClO_4_ to form (d) Pt/bTNT0, (e) Pt/bTNT5, and (f) Pt/bTNT30.

The overall morphology of different types of samples as observed by scanning electron microscopy (SEM) can be seen in Figure 1a–c. Except for the bTNT0 sample, which appears with the expected hexagonal, honeycomb‐like impressions left on the Ti foil from the bottom side of the peeled off nanotubes layer, the rest of the samples have the typical features of nanotubes grown in ethylene glycol‐based electrolytes.[^47^, ^48^] The top surface has the distinct honeycomb‐like morphology, with increasing pore diameter as the anodization times increased, and is free of any “nanograss” or other irregular features.^[49]^ Cross‐sectional images revealed close‐packed, vertically oriented, and relatively smooth nanotube surfaces of increasing lengths with increasing anodization times (Figure S1 in the Supporting Information). The geometrical parameters of the samples are summarized in Table S1 and are in good accordance with our previous studies.^[47]^

An estimate of the true projected area of the samples can be made by identifying the number of pores per unit area, calculating their area by means of the typical pore diameter and subtracting that from the nominal substrate area (see supplementary note 1 in the Supporting Information). Such an estimate gives a true geometric area that is 76 and 44 % of the projected nominal area for samples Pt/bTNT5 and Pt/bTNT30, respectively.

**Figure 2:**
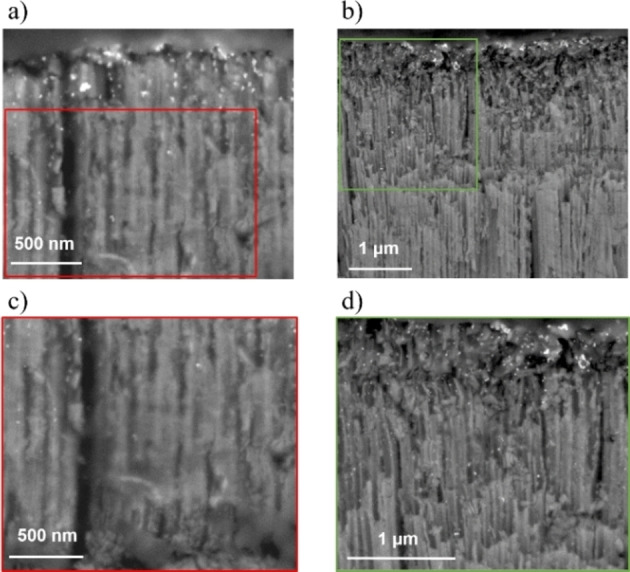
Cross‐section SEM images of (a,c) Pt/bTNT5 and (b,d) Pt/bTNT30, and higher magnifications in the designated areas. The formation of Pt deposits well inside the nanotubular morphology is highlighted. Additional cross sections showing the whole length of the Pt/bTNT5 and Pt/bTNT30 are given in Figure S1c,d.

After the galvanic deposition of Pt, the top‐view images revealed that the platinized samples were evenly decorated with Pt particle aggregates as it can be seen in Figure 1d–f. More importantly though, the interior wall of the nanotubes was also partly decorated with some Pt NPs, as it can be seen in Figure 2. From the cross‐sectional SEM images it appears that the Pt NPs have been deposited in a depth of approximately 0.8 and 4.2 μm from the opening of the nanotubes in the PtbTNT5 and PtbTNT30, respectively. The distribution of the galvanic deposits of Pt NPs on the walls of the nanotubes appeared graded, suggesting a diffusion‐controlled deposition.

**Figure 3:**
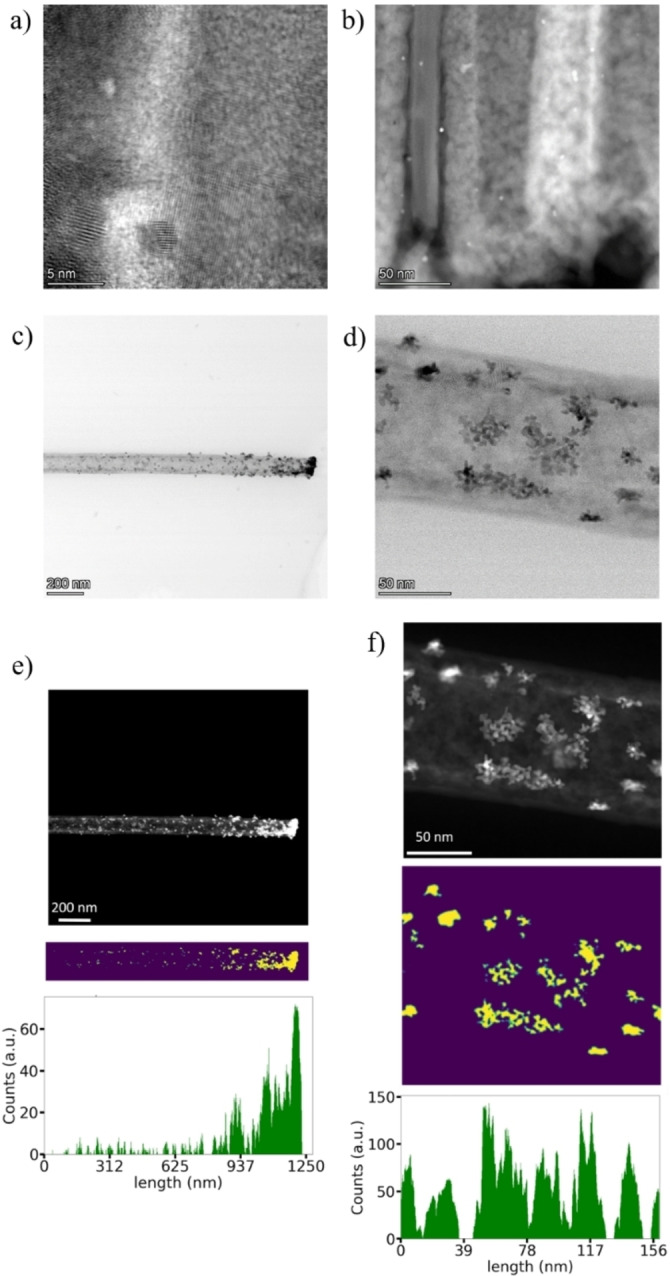
(a) High‐resolution STEM high‐angle annular dark‐field (HAADF) image of the Pt/bTNT30 depicting a Pt nanoparticle of around 2 nm and its interface with bTNT. (b) STEM HAADF image of the Pt/bTNT30 sample close to the bottom of a single nanotube. (c,d) STEM images of different magnifications of a single TiO_2_ nanotube from the Pt/bTNT30 sample, with a contour map of the bright pixels underneath. (e,f) The graphs show the number of bright pixels at each position in the *x* direction.

Scanning transmission electron microscopy (STEM) observations allowed to investigate at the nanoscale the electroless deposition of the Pt NPs on the interior wall of the nanotubes, as well as the depth range of the deposition. The Pt/bTNTs30 sample, which had the longest nanotubes, was observed, and Pt NPs with an average size of around 3.5 nm (Figure 3a) were detected all the way down to the bottom of the nanotubes (Figure 3b). The high crystallinity of the Pt NPs as well as its distinct interface with the crystalline TiO_2_ surface is revealed in the STEM images of Figure 3a. These results (see also Figure S2) show an intimate interface between the Pt nanoparticle and the TiO_2_ substrate. Lower magnification STEM images in Figure 3c,d underline the graded deposition of Pt along the nanotube axis. Close to the opening of the nanotube where the density of the particles is high, formation of Pt NPs aggregates is seen. It actually appears that these aggregates may be formed by a clustering of Pt NPs of around 2–3 nm (refer to Figure S3). This is interesting as these aggregates seem to provide a high catalytic surface area and moreover, the particle size matches the size of the Pt NPs deposited individually at the bottom of the nanotube. Furthermore, the interplanar distances match that of metallic Pt and the fast Fourier transform (FFT) suggests a cubic structure, as it can be seen in Figure S3c,d.

To better visualize the distribution of the Pt NPs a threshold was applied to the STEM images of Figure 3c,d, and the bright pixels of each column counted (Figure 3e,f). The result can be seen in the same figures. The maps below the STEM images show contour plots of the bright areas in the STEM image, with green marking the border and yellow the bright region. The graphs represent the number of bright pixels for each column. Figure 3e shows an entire nanotube, and a fluctuating, but ultimately decreasing number of bright pixels from right to left. Consequently, there is less Pt closer to the nanotube bottom than on the opening. Figure 3f shows a magnified region of Figure 3e and exemplifies that the distribution of brighter pixels across a short range varies significantly, while the mean distribution remains roughly constant. We can see a graded galvanic deposition of Pt from the opening and across the nanotube axis following an exponential dependence. This finding suggests that either the reactivity of bTiO_2_ decreases when approaching the bottom of the nanotube and/or the aqueous electrolyte displacement in the nanotube follows an exponential decay, assuming fast galvanic deposition kinetics. Another possibility could also be a depletion of the PtCl_6_^−2^ concentration along the nanotube axis strong capillary forces that can flood fast enough with solution these high aspect ratio structures. It is evident that nano‐fluidic studies utilizing our suggested methodology of in‐situ formation of nanoparticles in the interior wall of such high aspect ratio structures can be proven of significance, especially for applications in drug delivery, point‐of‐care testing, separation, sensors etc.[^50^, ^51^] We also used such contour plots to get a better overview on the size distribution of the Pt NPs from a larger sample area (i. e. additional STEM images). Figure S4 shows histograms of four STEM images from the Pt/bTNT30 sample, as well as their sum. As we can see from the total histogram, most of the NPs have a diameter between 1 and 5 nm with the maximum being at 2 nm.

**Figure 4:**
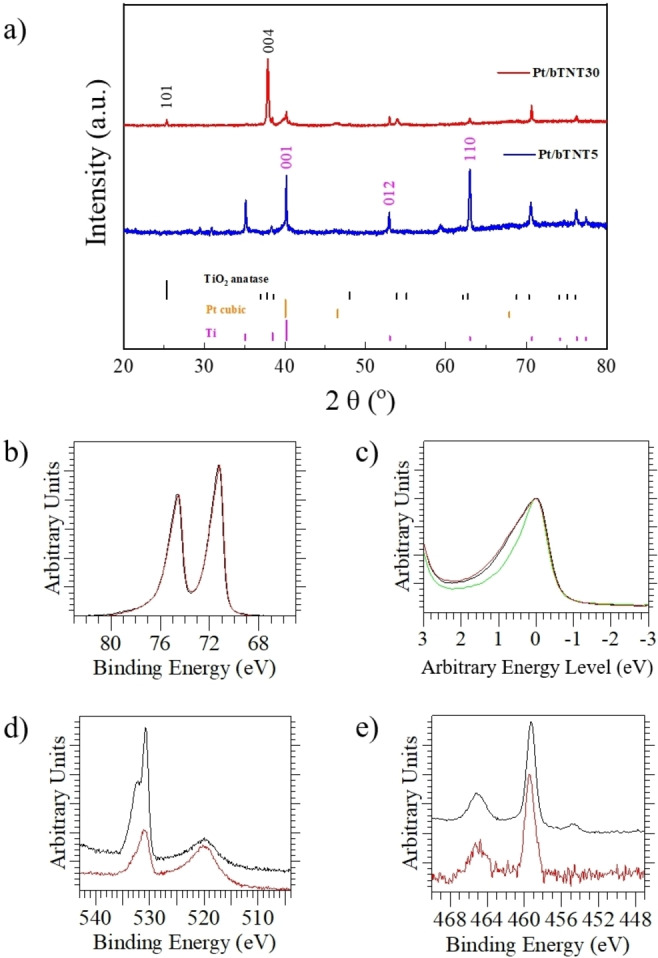
(a) XRD patterns of the Pt/bTNT5, and Pt/bTNT30 samples. The insets include high magnifications in the Miller indices of (101) and (004). The standard JCPDS cards of anatase TiO_2_ (red), Ti foil (pink), and cubic Pt (blue) are also given for reference. (b) Superimposed and normalized XPS spectra of Pt 4f_5/2_ and Pt 4f_7/2_ of the Pt/bTNT30 (dark red curve) and Pt/bTNT0 samples (black curve). (c) Pt 4f_7/2_ peaks superimposed and normalized for same intensity of the Pt/bTNT30 and Pt/bTNT0 samples as compared to pure Pt (green curve)‐peak maxima were placed arbitrarily at a 0 eV binding energy in order to compare the peak widths. (d) O 1 s peak and (e) Ti 2p_1/2_ and Ti 2p_3/2_ peaks.

The X‐ray diffraction (XRD) patterns of the different Pt/bTNT samples are presented in Figure 4a. The Pt/bTNT0 is not given for clarity of presentation, as both XRD diffractograms of the Pt/bTNT5 (short nanotubes of ≈2 μm) and Pt/bTNT0 (patterned Ti foil) samples were identical and contained mainly peaks from the Ti substrate and the Pt particles. In general, the main peaks were indexed according to the ICDD‐JCPD files for anatase, hexagonal Ti, and cubic Pt, the latter in agreement with the STEM results above, as it can be seen in the reference curves included in Figure 4a. The increased intensity of the (004) peak in the Pt/bTNT30 sample indicates the preferential crystal orientation of TiO_2_ along the tube growth direction. This is in agreement with our previous results, as we have shown that CaH_2_ annealing favors the preferential orientation towards the [004] direction.[^5^, ^52^] In general, the (101) surface of TiO_2_ dominates the external surface of anatase (more than 94 %),^[53]^ therefore the increased intensity of the (004) surface compared to the (101) one in our Pt/bTNT30 further highlights the strongly oriented nanotube structure.

X‐ray photoelectron spectroscopy (XPS) investigations were conducted in the Pt/bTNT0 and Pt/bTNT30 samples, as they represent the two limiting cases (patterned substrate and longest nanotubes, respectively). The superimposed and normalized XPS spectra of the Pt 4f_5/2_ and Pt 4f_7/2_ peaks (Figure 4b) at 74.5±0.1 and 71.2±0.1 eV show no essential differences in the line shape and the corresponding binding energies of the main peak, indicating no differences in the chemical state of Pt in these samples. Compared to the Pt 4f_7/2_ peak of a clean Pt standard (Figure 4c), both Pt/bTNT sample show slightly broader peak that may be attributed to chemisorbed O on Pt surface and/or PtO_*x*_.^[54]^ This is more clearly shown in the peak fitted spectra of Figure S5a, where the different oxidation states of Pt are also mentioned. The O 1 s peak at 530.7±0.1 eV (Figure 4d) corresponds to the lattice oxygen in TiO_2_, while the shoulder at the higher binding energy of 532.4±0.1 eV is related to oxygen‐deficient sites and/or surface −OH groups.[^55^, ^56^] As the difference in binding energies is approx. 1.7 eV this corresponds well with the presence of oxygen vacancies as molecular oxygen can be dissociatively adsorbed on the vacant sites ($O_{2}^{2-}$ , O^−^).^[56]^ A deconvolution of the 1 s peak (Figure S5b) shows the presence of an intermediate peak in the case of Pt/bTNT30, which can be attributed to adsorbed species and the ex‐situ type of the analysis. The peak at 519.9±0.1 eV is assigned to the Pt 4p_3/2_.^[57]^ Finally, the peaks at 465±0.2 eV and 459.4±0.2 eV correspond to Ti 2p_1/2_ and Ti 2p_3/2_ respectively of TiO_2_. A low intensity peak at 454.7±01 eV in the case of the Pt/bTNT0 sample can be assigned to metallic Ti (Ti^0^) and most probably related to the Ti foil under a thin bTiO_2_ film (Figure 4e). We have not identified any peak related to Ti^3+^ species in the Pt/bTNT30, but a slight possibility of such a presence is manifested by the Ti 2p_3/2_ peak broadening towards lower binding energies of the Pt/bTNT0 sample (see Figure S5c). However, as no distinct peak is shown in the spectrum of Pt/bTNT30, the presence of Ti^3+^ is considered insignificant a fact that is in agreement with our previously results and electron paramagnetic resonance (EPR) measurements, where no Ti^3+^ signals were observed (see also Figure S6).[^5^, ^58^]

### Electrochemical results

**Figure 5:**
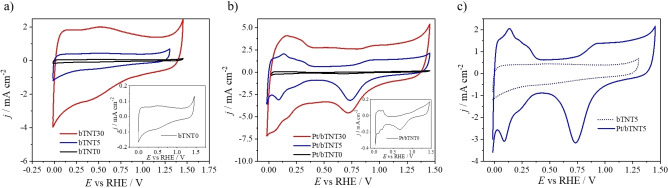
CV profiles of (a) bTNTs substrates, (b) Pt/bTNTs, and (c) Pt/bTNT5 and bTNT5 at 50 mV s^−1^ in deaerated 0.1 m HClO_4_.

The electrochemical behavior of three characteristic electrodes (Pt/bTNT0, Pt/bTNT5, and Pt/bTNT30 and their bTNT substrates) is depicted in the cyclic voltammetry (CV) profiles of Figure 5. As it can be seen in Figure 5a, bTNTs of the higher nanotube length and hence higher surface area have higher capacitive/pseudocapacitive currents, which is depicted as a higher capacitive (current) envelope of the voltammograms. The platinized bTNTs exhibit mixed surface electrochemistry of both Pt (see below H and O electrosorption/electrodesorption) and bTNT (large capacitive envelope), as it can be seen in Figure 5b that contains the CVs of all platinized bTNTs. This is more clear in Figure 5c, which presents the interfacial electrochemistry of Pt/bTNT5 and, for comparison, that of its respective substrate bTNT5.

Two sharp shaped and well distinguished reversible peaks attributed to strong and weak adsorption and desorption of underpotentially deposited hydrogen atoms (UPD−H) on the Pt particles appear at around +0.20 and +0.10 V_RHE_ (RHE: reversible hydrogen electrode). Pt oxide formation takes place at around +0.95 V_RHE_, while its reduction is shown at the distinctive peak of +0.73 V_RHE_. These typical Pt features are in accordance with the literature.^[38]^ At the same time, the characteristic feature of bTNT during the anodic scan at around +0.50 V_RHE_ (see also Figure 5a) and the high capacitive currents due to their high surface area are also observed‐superimposed in these CVs.

The electroactive area may be estimated from the UPD−H peaks of the CVs of the Pt/bTNT, as a monolayer of hydrogen atoms is adsorbed at Pt surface (corresponding to coverage of 210 μC cm^−2^). However, in this case, the high capacitive currents of the bTNTs, especially in the case of Pt/bTNT30, are likely to limit the accuracy of such an estimate. Nevertheless, carrying out this calculation an electroactive surface area (ESA) of 26.463, 28.553, and 1.464 cm^2^ per nominal cm^2^ has been estimated for electrodes Pt/bTNT30, Pt/bTNT5, and Pt/bTNT0, respectively. The fact that the optimum value of mass‐specific Pt electroactive area has been observed for the Pt/bTNT5 electrode could be explained by the fact that it contains much rougher deposits than the flat, patterned Pt/bTNT0 electrode (despite the latter having a larger true projected area and similar Pt loading). In contrast, isolated Pt nanoparticles within the long pores of the Pt/bTNT30 electrode may not be fully wetted or experience ohmic losses through the nanopores and/or bad electronic contact to the substrate.

### Hydrogen evolution studies

**Figure 6:**
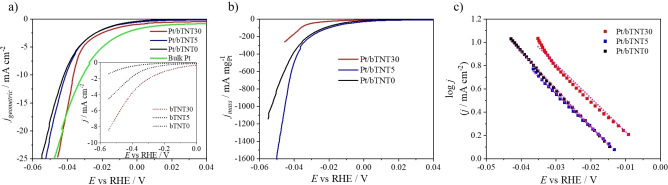
Polarization curves of *j* normalized (a) per Ti foil geometric area (inset shows the corresponding HER of the bTNT substrates) and (b) per Pt mass vs. *E* corrected for ohmic losses recorded by linear sweep voltammetry of Pt/bTNT and Pt bulk at 5 mV s^−1^ in deaerated 0.1 m HClO_4_. (c) Tafel plots of the Pt/bTNT.

Hydrogen evolution was studied by linear sweep voltammetry (LSV) at a slow scan rate of 5 mV s^−1^ in 0.1 m HClO_4,_ recorded from +0.30 V_RHE_ (potential range prior to hydrogen adsorption) up to −0.15 V_RHE_. Electrochemical impedance spectroscopy (EIS) spectra were recorded during HER to correct the potential for ohmic losses and, the solution ionic resistance was estimated at approximately 3.1–4.3 Ω. Figure 6a presents the hydrogen evolution performance of the Pt/bTNT and their respective substrates. The valuable contribution of Pt on the electrocatalytic performance of the platinized samples on HER is obvious when these are compared to their substrates, the latter requiring much higher overpotentials to achieve the same current. Note that in order to reach −10 mA cm^−2^ bTNT require at least 590 mV, whereas Pt/bTNT 36 mV.

Pt/bTNT30 exhibits enhanced apparent electrocatalytic performance (i. e., per nominal substrate electrode area) towards the HER compared to the rest of Pt/bTNT samples. This should be attributed to the higher overall Pt loading that has been deposited on this electrode in contrast to Pt/bTNT5 and Pt/bTNT0, as it will be mentioned below. Their apparent electrocatalytic activity towards HER is comparable to bulk Pt as it can be seen in Figure 6a. However, the bulk Pt performance still appears superior at low overpotential values, but at −10 mA cm^−2^ an overpotential of 31 mV for bulk Pt and 36 mV for Pt/bTNT30 is required. This may be due to the fact that highly active and porous electrodes are more likely to be blanketed or clogged by evolving hydrogen than a smooth and flat foil electrode.

Pt mass can be estimated by the inductively coupled plasma mass spectrometry (ICP‐MS) technique as described in the experimental section below. The results from the ICP‐MS analysis gave 94.38, 18.56, and 22.58 μg cm^−2^ Pt for Pt/bTNT30, Pt/bTNT5, and Pt/bTNT0 respectively. We can observe that a significantly greater amount of Pt has been deposited via the galvanic deposition process at the Pt/bTNT with the higher nanotube length (Pt/bTNT30) than at those with a shorter nanotube length (Pt/bTNT5) or the patterned electrodes (Pt/bTNT0). The fact that the short nanotube length electrode (Pt/bTNT5) exhibits a similar Pt loading to the patterned flat sample (Pt/bTNT0) may be attributed to the lower (44 % of its nominal value; see Experimental Section) true projected area of the former (where most deposition occurs), which is offset by a rougher deposit morphology and (limited) deposition into the pores. These loadings, together with the electroactive surface areas estimated by the hydrogen adsorption/desorption charge (see discussion of interfacial electrochemistry above) give Pt mass specific (electroactive) surface areas 28, 153, and 6.4 m^2^ g^−1^ for the Pt/bTNT30, Pt/bTNT5, and Pt/bTNT0 samples, respectively.

In Figure 6b Pt/bTNT electrodes are compared accounting for their Pt mass content. The Pt/bTNT5 electrode exhibits the highest per Pt mass performance. Similar Pt mass content has been deposited at Pt/bTNT0 and Pt/bTNT5, but Pt/bTNT5 gives slightly better results. This leads us to the conclusion that a length‐optimized nanotube substrate assists appropriate Pt deposition and dispersion and as a result enhances HER performance in comparison to the patterned substrate. However, the superiority of the patterned or short nanotube samples, as far as Pt mass specific activity is concerned, when compared to the long nanotube sample Pt/bTNT30 (despite the latter bearing a higher Pt loading), means that it is only the outer Pt layers that are active, while particles down the nanotubes are likely to be inoperative due to pore clogging during hydrogen evolution. Notice that the cross‐section SEM and the TEM images showed Pt particles deposition all the way down to the bottom of the nanotubes.

The HER is a two‐step process; the first step is the Volmer step [Eq. (1)], based on which a proton (H^+^) is reduced and adsorbed on the active site of catalyst surface as a H_ads_ atom and the subsequent second step involves the evolution of H_2_ gas. The second step follows either the H^+^ reduction on H_ads_ accompanied by an e^−^ transfer [Heyrovsky step; Eq. (2)] or the recombination of two H_ads_ [Tafel step; Eq. (3)] resulting at the formation of molecular H_2(g)_.[^37^, ^59^, ^60^, ^61^]

(1) $$\mathrm{Volmer}\;\mathrm{reaction}:\;H{}^{+}+e{}^{-}\to H{}_{\mathrm{ads}}$$

(2) $$\mathrm{Heyrovsky}\;\mathrm{reaction}:\;H{}_{\mathrm{ads}}+H{}^{+}+e{}^{-}\to H{}_{2(g)}$$

(3) $$\mathrm{Tafel}\;\mathrm{reaction}:\;2H{}_{\mathrm{ads}}\to H{}_{2(g)}$$

In our work, the Tafel slope is estimated to be 32, 34, and 30 mV dec^−1^ for Pt/bTNT30, Pt/bTNT5, and Pt/bTNT0 respectively (Figure 6c), corresponding to the same rate‐determining step of 30 mV dec^−1^ of bulk polycrystalline Pt at low overpotential values.[^37^, ^59^, ^62^] This value indicates that the reaction follows the Volmer–Tafel pathway as the rate‐determining step is the Tafel step. This means that the Pt‐bTNTs interactions do not affect/modify the mechanism of HER on Pt at the prepared electrodes.

In Table 1 data for the hydrogen evolution performance of similar electrodes are summarized in order to compare results that are found in the literature with the results of this work for Pt/bTNT30 and Pt/bTNT5. As it can be seen, the platinized black TNT electrodes of the present work (prepared via the galvanic deposition method) exhibits comparable apparent (per substrate nominal geometric area) HER performance with most titania‐based and carbon‐based electrodes that appear in the literature. At overpotential values other than the 30 mV for which values are shown in Table 1, commercial Pt/C (20 % Pt) electrocatalysts give 115 mA mg^−1^_Pt_ at an overvoltage of 15 mV,^[63]^ and 650 mA mg^−1^_Pt_ at that of 40 mV,^[64]^ while the catalysts of this work exhibit comparable mass performance of 20–76 mA mg^−1^_Pt_ at 15 mV and 172–500 mA mg^−1^_Pt_ at 40 mV, respectively.

**Table 1:** HER performance of platinized carbon and titania electrodes reported in the literature and this work.

Electrode	*η* ^[a]^ [mV]	*j* ^[b]^ [mA mg^−1^_Pt_]	HER conditions		Tafel slope [mV dec^−1^]	Ref.
Pt bulk	26	–	potential pulses, HClO_4_		31	[65]
Pt/C (20 %wt Pt) commercial	27	112.75	5 mV s^−1^, H_2_SO_4_		35	[66]
Pt/C (5 %wt Pt) commercial	50	137.25	5 mV s^−1^, H_2_SO_4_		40	[66]
Pt/graphite tubes; electrochemical deposition	18	–	2 mV s^−1^, H_2_SO_4_		24	[67]
Pt/graphite tubes; electrochemical deposition	66	–	2 mV s^−1^, HClO_4_		24	[67]
Pt/C	81	19.64	2 mV s^−1^, HClO_4_		30.4	[67]
Pt/C commercial	44.17	224.7	20 mV s^−1^, H_2_SO_4_		–	[68]
Pt/N_*x*_:TiO_2‐*x*_; borohydride reduction	40	157	10 mV s^−1^, H_2_SO_4_		33	[39]
($v_{O}$ ‐rich Pt/TiO_2_)/GC	60	1250	5 mV s^−1^, H_2_SO_4_		33	[42]
($v_{O}$ ‐deficient Pt/TiO_2_)/GC	160	–	5 mV s^−1^, H_2_SO_4_		102	[42]
Pt/bTNT30; galvanic deposition	36	49	5 mV s^−1^, HClO_4_		32	this work
Pt/bTNT5; galvanic deposition	41	206	5 mV s^−1^, HClO_4_		34	this work

[a] Overpotential values required to reach current density of −10 mA cm^−2^. [b] Current density per Pt mass at an overvoltage of 30 mV.

Stability testing of the Pt/bTNT30 sample was performed under galvanostatic conditions at −10 mA cm^−2^ for 48 h at the same experimental acidic conditions of 0.1 m HClO_4_. Figure S7 shows that after 48 h of operation the potential showed a minimal increase, clearly indicating the high stability of the platinized bTNT. Post‐operation energy‐dispersive X‐ray spectroscopy (EDS) analysis of the electrode showed no change at the Pt content, which further indicates the good stability during HER performance. The stability tests were also carried out in a Nafion‐membrane separated two‐compartment cell with no detectable change observed during the course of the experiment (not presented).

### Galvanic deposition mechanism

The initial assumption behind the deposition mechanism included the oxidation of reduced surface species of bTiO_2_ by Pt(IV) cations. The kinetic force behind the galvanic/electroless deposition was considered to lie in the positive difference between the standard reduction potentials of TiO_2_ and Pt(IV). In our case the reduced surface species on TiO_2_ are neutral oxygen vacancies as Ti^3+^ is apparently not present according to our XPS and EPR measurements. In order to further elucidate the deposition mechanism, first‐principles calculations were considered using DFT.

**Figure 7:**
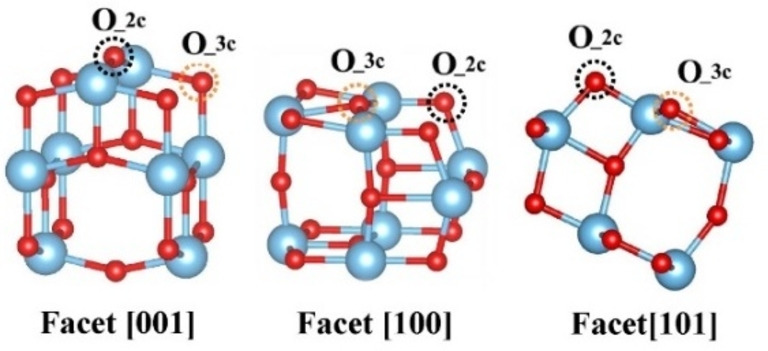
Surface structures of anatase TiO_2_ with different facets [001], [100], and [101].

The optimized TiO_2_ surface structures with three different facets [001], [100], and [101] are displayed in Figure 7. There are two types of oxides ion sites for all three facets (marked as O_2c and O_3c), yielding two types of oxygen vacancies correspondingly. For both types of neutral oxygen vacancies ($v_{O}^{\times }$ ), they are found to exist as defect complexes composed of the effective positively charged defect ($v_{O}^{••}$ ) and two electron polarons at the adjacent Ti atoms (${Ti}_{Ti}^{/}$ ). These electronic defects (${Ti}_{Ti}^{/}$ ) create in‐gap states and thereby extend the light adsorption in the near‐infrared region, rationalizing the black color of the anatase TiO_2_.

The defect formation energies of $v_{O}^{\times }$ are summarized in Table 2. For each crystal facet, the formation energy of $v_{O}^{\times }$ _2c is significantly lower than that of $v_{O}^{\times }$ _3c, showing the strong site preference of $v_{O}^{\times }$ formation. Meanwhile, the formation energy of $v_{O}^{\times }$ _2c also exhibits facet dependency, that is, the formation energy on facet [001] is significantly lower than those on [100] and [101] facets. These computational results indicate that the [001]‐oriented TiO_2_ promotes the formation of oxygen vacancies under the imposed reducing conditions. Thus, our facile method for the preparation of black TiO_2_ with tunable crystal structure is advantageous and matches perfectly with the simple galvanic deposition method for the deposition of high‐performance catalyst of low loadings (μg cm^−2^ range).

**Table 2:** Formation energies of neutral oxygen vacancy under dry H_2_ (H_2_=10 bar and H_2_O=10^−5^ bar) at 500 °C.

Oxide ion site	Formation energy [eV]		
	Facet [001]	Facet [100]	Facet [101]
$v_{O}^{\times }$ _2c	−1.55	−0.63	0.40
$v_{O}^{\times }$ _3c	0.70	0.28	1.09

To explore the formation process of Pt particles on TiO_2_, we calculated the adsorption energy of single Pt atom on the different facets. It is also assumed that the adsorbed Pt atom can act as the nuclei for the Pt particles growth. The Pt adsorption energies are summarized in Table 3.

**Table 3:** Adsorption energies of Pt atom at different TiO_2_ surfaces.

Surface type	Pt adsorption energy [eV]		
	Facet [001]	Facet [100]	Facet [101]
pristine surface	−1.75	−2.02	−1.02
surface with $v_{O}^{\times }\_2c$	−3.39	−3.04	−1.22

**Figure 8:**
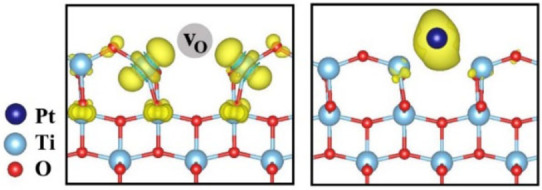
Isosurface of neutral oxygen vacancies ($v_{O}^{\times }$ ) and a single Pt atom adsorption on $v_{O}^{\times }$ .

It is found that the Pt adsorption energies on oxygen‐deficient surface are significantly lower compared to that on the pristine surface, showing that the defective surface favors the Pt adsorption on the surface. Among the three crystal facets studied and considered herein, the Pt adsorption energy on oxygen‐deficient [001] facet was the lowest compared to that of other two facets with a value of −3.39 eV. Furthermore, an analysis of the charge distribution clearly indicated that the electrons transfer from $v_{O}^{\times }$ towards the Pt atom during the adsorption process (Figure 8). The excess of electrons on Pt may additionally promote the reduction of the next adsorbed Pt ions, promoting the growth of Pt particles. Pt deposition on bTNT can be described with the following half‐reaction [Eq. (4)], based on which Pt(IV) is reduced as metallic Pt:

(4) $$\mathrm{PtCl}{}_{6}{}^{2-}+2\;\left(2e{}^{-}+v_{O}^{••}\right)\to \mathrm{Pt}+2v_{O}^{••}+6\;\mathrm{Cl}{}^{-}$$

where the positively charged oxygen vacancy $v_{O}^{••}$ and e‐ stand for the neutrally charged oxygen vacancy$\;\left(v_{O}^{\times }\right)$ . Finally, it is worth adding that the galvanic replacement process involves a partial surface oxidation of bTNT at locations nearby to those of Pt deposition. However, the rest of the material is expected to be unaffected because the oxide ion mobility at room temperature is very low, therefore the bulk of TiO_2_ can still maintain the high oxygen deficiency. Moreover, the loss of electrons during Pt deposition is compensated during operation as cathode. These assumptions are macroscopically confirmed by the clear, not distorted by ohmic losses electrochemistry.

## Conclusion

In this work and for the first time, we showed the deposition of Pt nanoparticles (NPs) across the whole length of high aspect ratio black TiO_2_ nanotubes. This was achieved by an in‐situ formation and deposition of the Pt NPs, facilitated by the galvanic deposition method. Our density functional theory studies elucidated the synergy between the preferential [004] orientation and the in‐situ formation of Pt NPs. Our results suggested that localized electrons at the oxygen vacancy sites can reduce Pt(IV) ions from an aqueous electrolyte solution and deposit Pt NPs of approximately 2.5 nm. This mechanism and formation of Pt NPs are favored in the [004] oriented nanotubes and an exponential deposition profile of Pt NPs along the black TiO_2_‐nanotubes (bTNTs) has been observed by high‐resolution scanning transmission electron microscopy analysis. Meanwhile, inductively coupled plasma mass spectrometry measurements found that the mass loadings of Pt was in the range of a few tens of μg cm^−2^. The prepared platinized bTNT samples were tested as hydrogen evolution cathodes exhibiting high hydrogen evolution reaction (HER) performances, comparable to state‐of‐the‐art Pt‐based cathodes. Their performance could be tuned by varying the nanotube length, and the best results per substrate nominal geometric area were obtained for platinized nanotubes of a length of 10 μm, whereas those per mg of Pt were obtained for 2 μm long nanotubes. Finally, the good stability of the platinized bTNT has been witnessed by the minimal potential change in a 48 h long galvanostatic experiment. This work contributes to the preparation of highly active HER cathodes based on noble metal catalysts of low mass loadings by minimal engineering. Moreover, this facile method of in‐situ NPs formation can assist in nano‐fluidic studies in high‐aspect‐ratio nanostructured materials with applications in drug delivery, sensors, membranes, and others. Finally, we provided important insights on the underlying deposition mechanism, correlated to tunable structural and electronic properties.

## Experimental Section

### Chemicals and apparatus

Ti sheet (0.25 mm thick, 99.7 % purity), ethylene glycol (analytical grade), and ammonium fluoride (analytical grade) were purchased from Sigma‐Aldrich. The DC power supply used to prepare titania nanotubes was by Keithley Instruments (Model 2200–72‐1DC). Calcium hydride (CaH_2_, ≥97.0 % powder) and potassium hexachloroplatinate (K_2_PtCl_6_, ≥99.9 % trace metals basis) were supplied from Sigma Aldrich, perchloric acid (HClO_4_, 70 %) from Merck, and hydrochloric acid (HCl, 37 % for laboratory use) and nitric acid (HNO_3_, 65 %) from ChemLab.

### Preparation of the Pt/bTNT electrocatalysts

The synthesis of the Pt/bTNT electrocatalysts included the preparation of the bTNT and the Pt deposition on them via the spontaneous galvanic deposition method. Self‐ordered TNT was synthesized by the well‐established two‐step anodization process of Ti foils. Before anodization, the Ti foils (1 cm×1 cm) were ultasonicated in isopropanol, acetone, and then water for 15 min in each solution and left to dry vertically in air. Ti foils were anodized applying a voltage of 60 V by a DC power supply, in an organic ethylene glycol solution containing 0.25 wt % NH_4_F and 2 wt % H_2_O, in a two‐electrode system. Each Ti foil was placed at a constant distance of 2 cm from a Pt foil, used as the counter electrode. Ti foils were anodized in two anodization steps. The first anodization step lasted 90 min and the oxide layer was removed, revealing a TNT pattern on the Ti surface. During the second step the patterned Ti substrate was subjected to the anodization process in a fresh solution for a further period of time to create stable, self‐ordered, and well‐structured titania nanotubes. The growth of the titania nanotubes was assisted by the already formed TNT pattern on the Ti substrate. Varying the duration of the second anodization step resulted in different TNT lengths, with 30, 5, and 0 min corresponding to 10, 2, and 0 μm length of the titania nanotubes, as reported in a previous work.^[47]^ Note that samples of 0 μm length correspond to the patterned Ti substrates. The TNT/Ti foils were left in the same solution for at least 1 h to improve nanotube adhesion. The thus prepared TNT/Ti foils were placed in a quartz tube in direct contact with CaH_2_ and the tube was sealed under vacuum and then placed in a furnace to anneal the samples at 500 °C for 2 h (with heating and cooling rates of 1.6 °C min^−1^). The TNTs obtained anatase structure at 500 °C and metallic‐like conductivity at the reducing atmosphere resulted in conductive bTNT (refer to our electrochemical impedance data in our previous works[^5^, ^52^]). In this work these bTNT samples are referred as bTNT30, bTNT5, and bTNT0, respectively, based on the second step anodization time.

The deposition of metallic Pt was achieved by the galvanic deposition method. The sealed quartz tube was opened and the freshly reduced and completely dry specimens were immersed immediately in a Pt(IV) ion complex solution. This step ensures that the complex solution fills the TNT pores and the Pt deposition can happen all the way to the bottom of the nanotube. The N_2_‐deaerated Pt(IV) solution contained 0.5 mm K_2_PtCl_6_ and 0.1 m HClO_4_. The galvanic deposition took place at 65 °C for 10 min under a continuous N_2_ flow above the solution. The chloroplatinate solution was deoxygenated with N_2_ gas to avoid O_2_ reduction instead of Pt(IV) reduction, as O_2_ reduction is a thermodynamically competitive reaction.

### Microscopic and spectroscopic analyses

SEM (Hitachi SU8230, an acceleration voltage of 3 kV) equipped with EDS was used to analyze the overall morphology and composition of the prepared electrodes. The cross‐sectional SEM images were obtained after polishing of epoxy resin‐embedded samples in order to obtain true images of the interior of the nanotubes. STEM was performed with a Titan G2 60–300 instrument, operated at 300 kV with 80 pA beam current and 0.08 nm of nominal spatial resolution, to obtain the morphology of single nanotubes and Pt nanoparticles. The samples were investigated using data collected by annular bright‐field (ABF), low‐angle annular dark field (ADF), and high‐angle annular dark field (HAADF) detectors. The crystalline phase of the samples was identified by XRD (Bruker D8 Discover, Cu K_α1_‐filters radiation, *λ*=1.5406 Å, Bragg–Brentano) with a 0.02° (2*θ*) step, and dwell time of 10 s step^−1^. For the identification of the chemical state of the sample surfaces, XPS was performed on a KRATOS Axis Ultra^DLD^ using monochromatic Al K_α_ radiation (1486.6 eV). Survey and high‐resolution scans were acquired at the pass energies of 160 and 20 eV, respectively. All spectra were acquired at a 0° angle of emission (vertical emission). The casaXPS software was used for data processing. The Pt mass loading was determined by ICP‐MS, at a Thermoscientific iCAP Q ICP‐MS controlled by Q Tegra Software. Each sample was subjected to chemical digestion in 4 mL aqua regia (HCl+HNO_3_) for 30 min. The leachates were diluted in 2 % HNO_3_ before measurement.

### Electrochemical setup

The electrochemical experiments were conducted in a three‐electrode system to study the platinized bTNTs and their corresponding substrates. A Pt foil was used as the counter electrode and a saturated calomel electrode (SCE sat. KCl) as the reference electrode. All the referred potentials in the plots of this work are quoted versus RHE (reversible hydrogen electrode) based on the following Equation (5) (applied for 0.1 m HClO_4_ and pH=1:

(5) $$E{}_{\mathrm{RHE}}=E{}_{\mathrm{SCE}}+E{}^{0}{}_{\mathrm{SCE}}+0.059\;\mathrm{pH}=E{}_{\mathrm{SCE}}+0.244+0.059\;V=\;E{}_{\mathrm{SCE}}+0.303\;V$$

Note that the equilibrium potential for the hydrogen evolution reaction in 0.1 m HClO_4_ at room temperature is shifted at −0.303 V vs. SCE and is 0 V vs. RHE. Hence, the applied potential referring to RHE and the overpotential coincide in the case of HER.

The electrochemical behavior was studied by CV between the onset of hydrogen and oxygen evolution reactions at a potential scan rate of 50 mV s^−1^ in 0.1 m HClO_4_. HER was studied by near‐steady‐state linear sweep voltammetry (LSV) carried out at a low scan rate of 5 mV s^−1^ up to −0.45 V_SCE_ in 0.1 m HClO_4_. After the HER study, impedance curves were recorded to determine the resistance of the solution at high frequencies in order to correct the potential for ohmic losses. EIS experiments were performed in the frequency range between 100 kHz and 100 mHz in the DC potential range of −0.3 to −0.36 V_SCE_ in 20 mV intervals, recording alternate current response at an AC voltage amplitude of 10 mV, in order to correct the uncompensated resistance during the HER. The electrochemical experiments were conducted with an Ivium Vertex potentiostat/galvanostat (Ivium Technologies) controlled by Ivium Software. Also, evaluation of the electrocatalyst stability was carried out at −10 mA cm^−2^ in 0.1 m HClO_4_, recording the potential response during 48 h. All electrochemical studies were carried out at room temperature (≃20 °C). Finally, in order to compare the platinized bTNTs with the state‐of‐the‐art HER electrocatalyst, a bulk Pt was scanned at the same conditions using a Pt rotating disk electrode at 1500 rpm in order to avoid H_2_ gas blocking of its horizontally placed surface.

### Computational methods

First‐principles calculations were performed using DFT as implemented in the VASP code.[^69^, ^70^] The revised Perdew‐Burke‐Ernzerhof (RPBE) exchange‐correlation functional^[71]^ was used throughout to describe exchange and correlation with a Hubbard‐U correction (GGA+U) of 3 eV to Ti 3d orbitals. This U value was determined by using the piece‐wise linearity methodology^[72]^ for a single localized electron in bulk anatase TiO_2_. The electronic wave functions were described using a plane‐wave basis set with an energy cutoff of 500 eV. The anatase TiO_2_ with different facet [001], [100], and [101] were represented as p(3×3), p(3×1), and p(2×3) slabs, respectively. The neighboring slabs were separated in the direction perpendicular to the surface with a vacuum layer of 16 Å and a 2×2×1 Γ‐centered k‐mesh was used for all calculations.

The formation energies of neutral oxygen vacancies ($v_{O}^{\times }$ ) were calculated according to Equation 6:^[73]^

(6) $${\Delta E}_{v_{O}}^{f}=E_{v_{O}}^{tot}-\;E_{Perfect}^{tot}+\mu_{o}$$

where $E_{v_{O}}^{tot}$ is the total energy of a supercell with neutral oxygen vacancy, while $E_{Perfect}^{tot}$ represents the total energy of the host supercell. As the experiments were performed by reducing TiO_2_ with the CaH_2_ powder, the chemical potential of$\mu_{o}$ was calculated through $\mu_{O}=\;\mu_{H_{2}O}-\mu_{H_{2}}$ under a dry reducing condition (H_2_=10 bar, H_2_O=10^−5^ bar.) The chemical potentials of $\mu_{H_{2}}$ and $\mu_{H_{2}O}$ are calculated as following [Eq. 7]:

(7) $$\mu \left(p,T\right)=E_{0}+\;H^{\theta }\left(T\right)-TS^{\theta }\left(T\right)+RT\ln\frac{p}{p^{\theta }}$$

$H^{\theta }\left(T\right)$ and $S^{\theta }\left(T\right)$ of the gas phases are taken from tabulated data.^[74]^

The adsorption energies (${\Delta E}_{ad}$ ) for a Pt atom adsorbed on the different TiO_2_ surfaces were calculated by Equation 8:

(8) $${\Delta E}_{ad}=E_{Pt/TiO_{2}}-\left(\;E_{{TiO}_{2}}+E_{Pt}\right)$$

where $E_{Pt}$ ,$\;E_{{TiO}_{2}}$ , and $E_{Pt/TiO_{2}}$ are the total energies of the individual Pt atom, TiO_2_ substrate, and Pt/TiO_2_ cluster, respectively.

Finally, the gradient in amount of Pt on a single nanotube was graphically represented by applying a threshold to a dark field image. The intensity of Pt is much higher than the intensity of the TiO_2_ tube in a dark field TEM image. By determining the number of high intensity pixels for each column of the dark field image, the distribution of Pt on the TiO_2_ nanotube can be determined. To perform this, a python code utilizing hyperspy was used.^[75]^

## Author Contributions

A. Touni investigation, formal analysis, methodology, writing – original draft; X. Liu data curation, formal analysis, investigation, methodology; X. Kang investigation; P. A. Carvalho investigation, funding acquisition, S. Diplas investigation, K. Both, investigation, data curation, formal analysis; S. Sotiropoulos conceptualization, supervision, validation, funding acquisition, writing review and editing; A. Chatzitakis formal analysis, investigation, methodology, project administration, resources, supervision, validation, funding acquisition, writing‐review and editing.

## Conflict of interest

The authors declare no conflict of interest.

## Supporting information

As a service to our authors and readers, this journal provides supporting information supplied by the authors. Such materials are peer reviewed and may be re‐organized for online delivery, but are not copy‐edited or typeset. Technical support issues arising from supporting information (other than missing files) should be addressed to the authors.

Supporting Information
