# Supplementary material for: Galvanic Deposition of Pt Nanoparticles on Black TiO2 Nanotubes for Hydrogen Evolving Cathodes
Source: ChemSusChem. 2021 Oct 6;14(22):4993–5003. doi: 10.1002/cssc.202101559 (PMC9291612; doi:10.1002/cssc.202101559)
Supplement: Supplementary file 1 — Supporting Information [file CSSC-14-4993-s001.pdf]

ChemSusChem

Supporting Information

Galvanic Deposition of Pt Nanoparticles on Black TiO_2 Nanotubes for Hydrogen Evolving Cathodes

Aikaterini Touni, Xin Liu, Xiaolan Kang, Patricia A. Carvalho, Spyros Diplas, Kevin G. Both, Sotirios Sotiropoulos,* and Athanasios Chatzitakis*© 2021 The Authors. ChemSusChem published by Wiley-VCH GmbH. This is an open access article under the terms of the Creative Commons Attribution License, which permits use, distribution and reproduction in any medium, provided the original work is properly cited.

List of figures

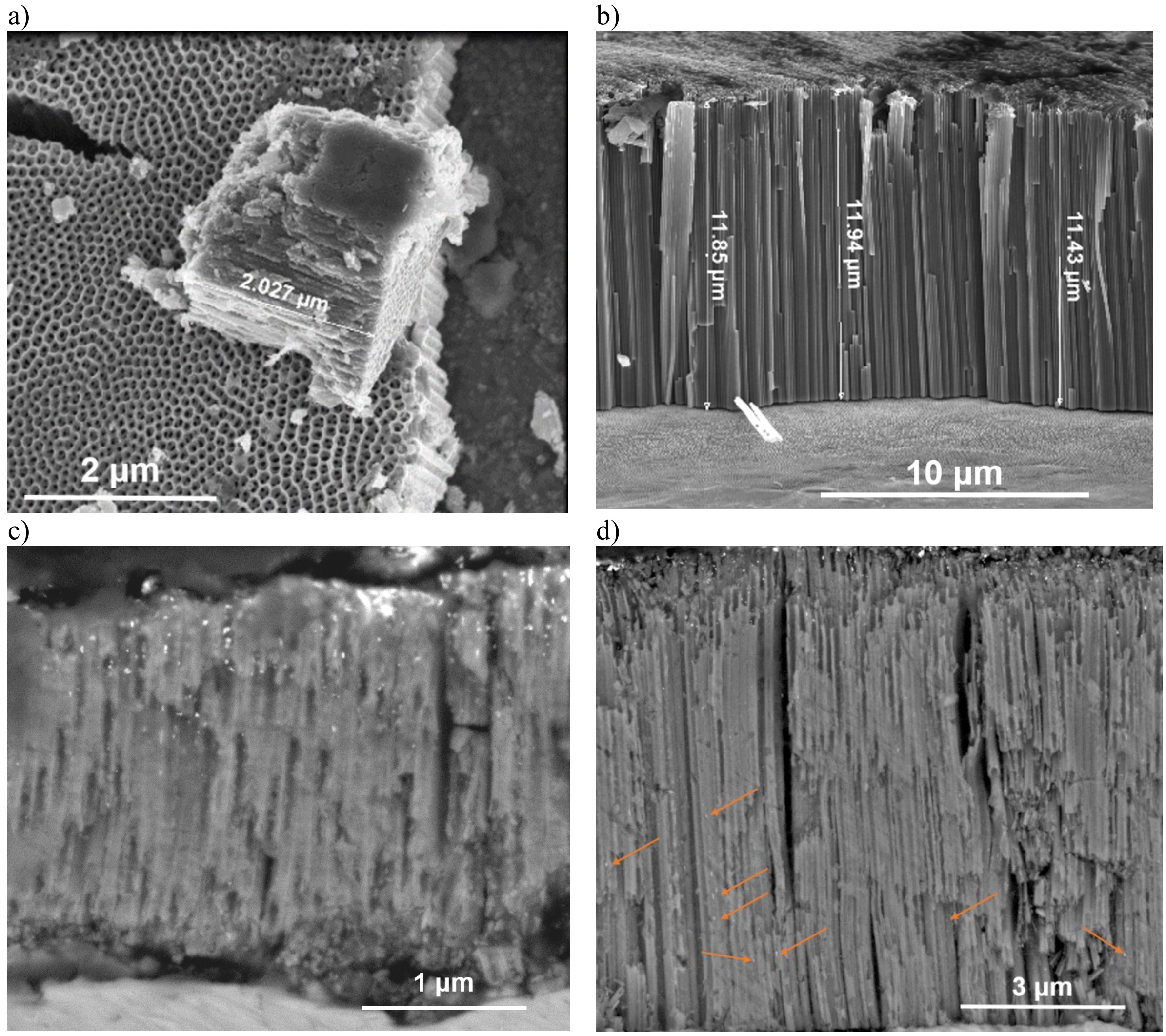

Figure S1: Cross sectional images of a) bTNT5, b) bTNT30, c) Pt/bTNT5 and d) Pt/bTNT30, where some Pt NPs are indicated with orange arrows.

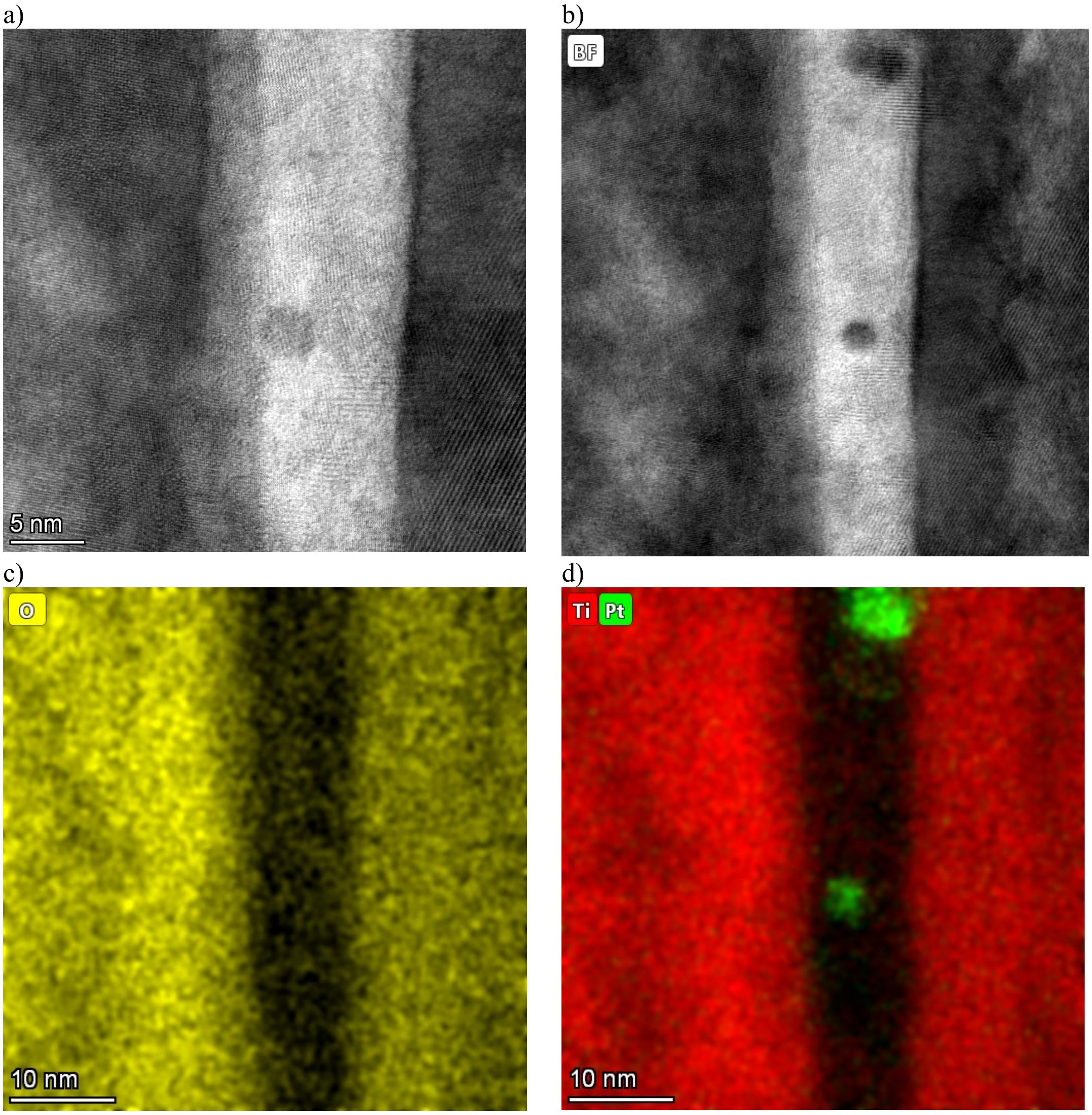

Figure S2: a) and b) HR STEM images of the Pt/bTNT30 sample depicting Pt nanoparticles of around 2 nm and their interface with the bTNT wall. c) EDS mapping of O, and d) Pt and Ti of image b.

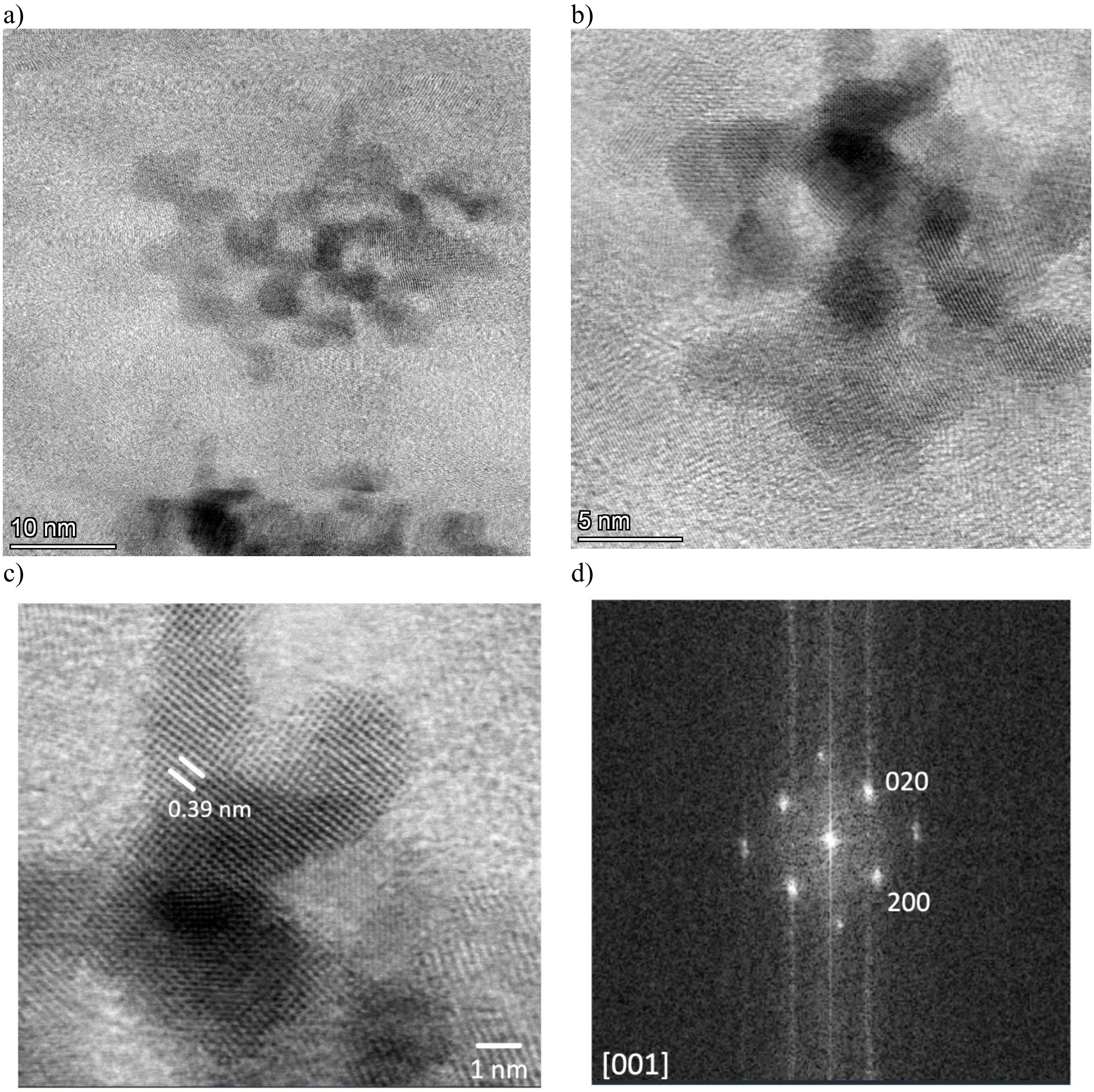

Figure S3: a) and b) HR STEM images of different magnifications of a Pt cluster close to the opening of the TiO_2 nanotube. c) Bright-field HRSTEM image and d) FFT of the top particle in a cubic orientation. The interplanar distances match metallic Pt. The magnifications were taken on the image in Figure 3d in the main document.

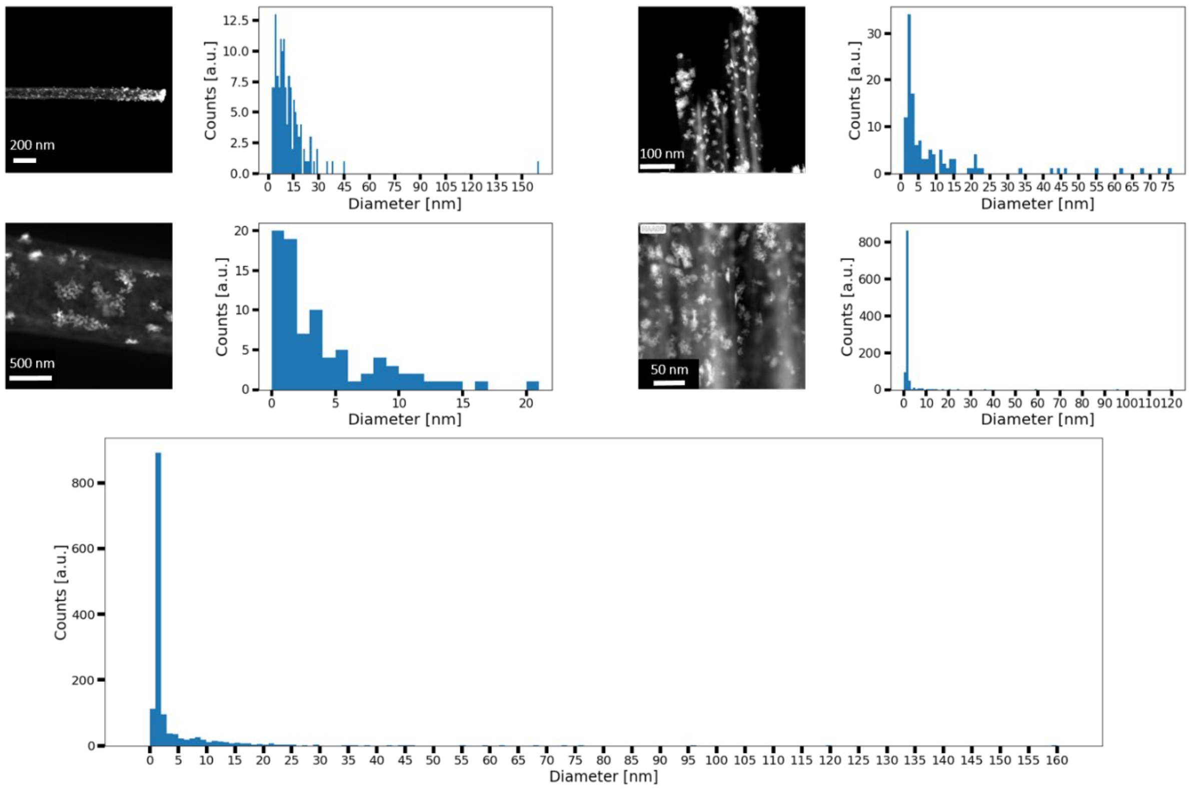

Figure S4: Pt NPs size distribution histograms. The images c) and d) are higher magnification images of a) and b) in order to analyse the distribution of the Pt NPs in the larger aggregates. The total size distribution of these four histograms are summarized in e). The area of each distinguishable shape was assumed to be circular, and the corresponding diameter extracted and plotted in a histogram. This assumption is true for small particles; however, it fails to represent the large ones accurately. The low number of large particles justifies the use of the method. It is easily observable that the majorities of diameters are less than 5 nm. However, evidently, there are some outliers, the biggest one with a diameter of 160 nm.

a)

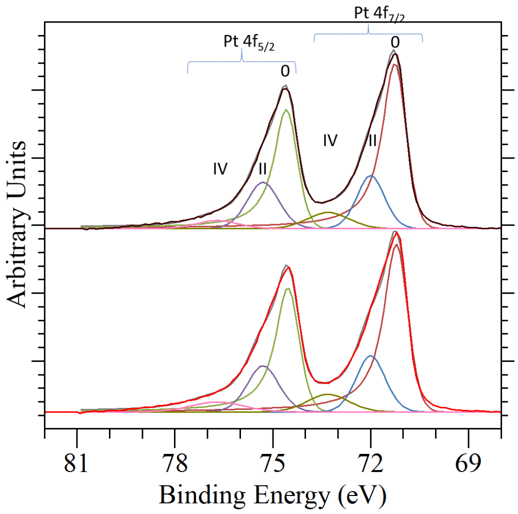

b)

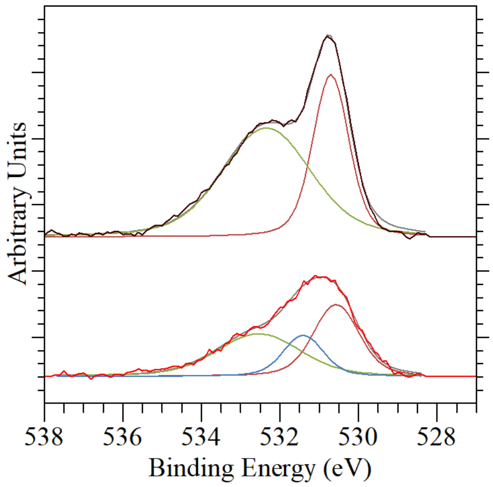

c)

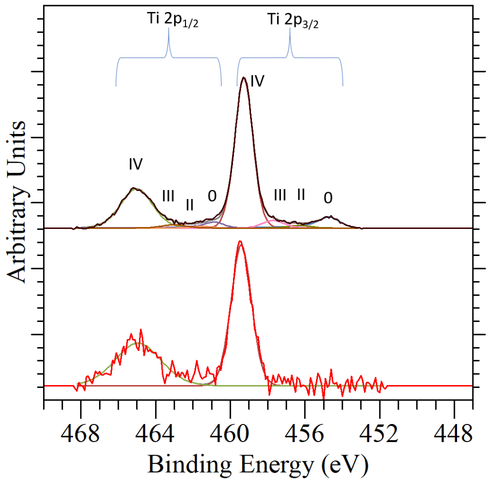

Figure S5: Peak fitted XPS spectra of a) Pt $4f_{5/2}$ and $4f_{7/2}$, b) O 1s and c) Ti $2p_{1/2}$ and Ti $2p_{3/2}$ signals indicating the additional chemical states in the Pt/bTNT0 (black curve) and Pt/bTNT30 (red curve) samples.

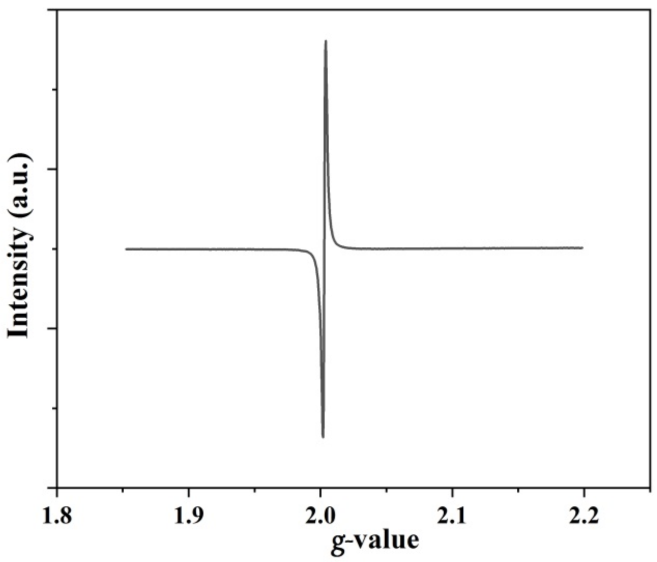

Figure S6: EPR spectra of bTNT, reproduced from our previous work in [1].

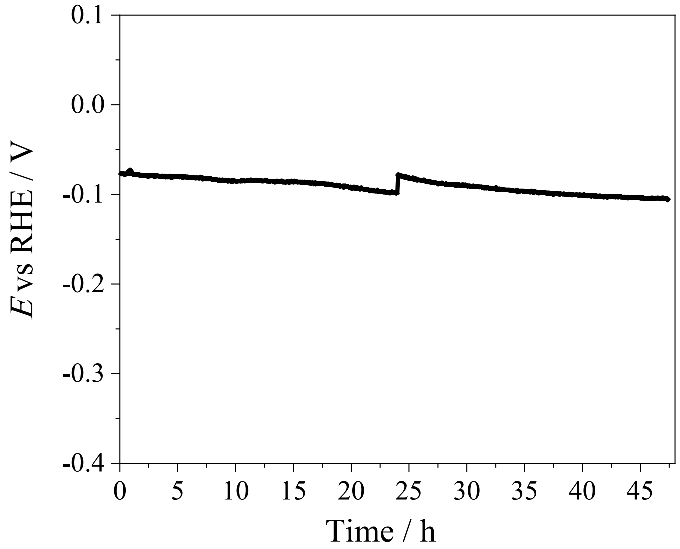

Figure S7: Stability testing under galvanostatic conditions at -10 mA cm^{-2} in 0.1 M $HClO_4$, with no IR correction of the Pt/bTNT30.

List of tables

Table S1: Average geometrical parameters of bTNT of different lengths as measured by the SEM images of Fig. S1 and Fig. 1 (b, c).

Sample	Length (nm)	Average Pore size (nm)
TNT5	2030	69
TNT30	11740	92

Supplementary note 1

This is a rough estimate of the projected area (the area of the rings of the nanotubes on top of the samples). We used SEM images of the top of the samples. An area of x nm × x nm contains y rings with a diameter of z nm (which is the average pore size of Table S1). The area of one pore is $\pi(z/2)^2$. The projected area equals x nm × x nm – y × $\pi(z/2)^2$ So the % ratio of the (true) projected area to the nominal substrate area is projected area / (x nm × x nm).

References

1 Liu, X.; Carvalho, P.; Getz, M. N.; Norby, T.; Chatzitakis, A. Black Anatase TiO2 Nanotubes with Tunable Orientation for High Performance Supercapacitors. J. Phys. Chem. C **2019**, 123 (36), 21931–21940.
